# In Situ EC‐EPR Spectroscopy and DFT Analysis of H_UPD_ on Polycrystalline Pt

**DOI:** 10.1002/cssc.202501908

**Published:** 2026-03-08

**Authors:** Rainer Götz, Kimmo Pyyhtiä, Bingxin Li, Theophilus K. Sarpey, Kun‐Ting Song, Mira Todorova, Nadezhda Kukharchyk, Siegfried Schreier, Pekka Peljo, Elena L. Gubanova, Jörg Neugebauer, Aliaksandr S. Bandarenka

**Affiliations:** ^1^ Physics of Energy Conversion and Storage TUM School of Natural Sciences Department of Physics Technical University of Munich Garching Germany; ^2^ Department of Mechanical and Materials Engineering University of Turku Turku Finland; ^3^ Max‐Planck‐Institut für Eisenforschung GmbH Düsseldorf Germany; ^4^ GSI Helmholtzzentrum für Schwerionenforschung GmbH Darmstadt Germany; ^5^ Walther‐Meißner‐Institute for Low Temperature Research Bavarian Academy of Sciences and Humanities Garching Germany; ^6^ Catalysis Research Center TUM Technical University of Munich Garching Germany

**Keywords:** electroadsorbed hydrogen, electrocatalysis, electron paramagnetic resonance spectroscopy, hydrogen adsorption sites, platinum, proton electroreduction

## Abstract

Electrochemical hydrogen production and conversion using renewable energy sources have become a key topic in catalysis research. Platinum and Pt‐group metals are among the best materials promoting H_2_ evolution (HER) and oxidation (HOR) reactions. However, the nature of active surface sites should be further elucidated to improve their performance and gain a better fundamental understanding of those processes. This is not a trivial task, mainly due to the high surface mobility of the H‐species. Here, we use in situ electron paramagnetic resonance (EPR) spectroscopy to investigate the Pt surface in the so‐called underpotential deposition (UPD) region in acidic media and observe EPR responses indicative of hydrogen adsorption sites, the knowledge of which is essential for both HOR and HER. Our EPR measurements and theoretical ab initio molecular dynamics (AIMD) calculations suggest that the average adsorption sites for atomic hydrogen at the surface of platinum are either on‐top sites or 3‐fold hollow sites, while bridge sites are not likely to be occupied. For EPR, the intensity maximum is reached at −0.85 V versus Pt, and then the signal intensity vanishes for potentials just before HER, suggesting EPR‐silent H_2_ formation. At the same time, ab initio density functional theory (DFT) calculations of a Pt(111) surface with 7/12 ML coverage of H at room temperature yield occupancy probabilities of 0.72 (fcc hollow), 0.26 (on‐top), and 0 (bridge) for the respective sites. Hence, fcc hollow is favored over on‐top adsorption sites at high coverages, which is consistent with the observation via EPR spectroscopy. To our knowledge, EPR spectroscopy was used for the first time to probe the EPR response during hydrogen electrosorption in the H_UPD_ region at polycrystalline platinum electrodes in acidic electrolytes.

## Graphical abstract

Electrochemical electron paramagnetic resonance (EC‐EPR) spectroscopy provides a more comprehensive view of hydrogen adsorption modes on polycrystalline Pt within H_UPD_ potentials. The results rule out bridge‐site adsorption, consistent with the accompanying ab initio molecular dynamics (AIMD) simulation of a high‐coverage Pt surface. The simulations further indicate a preference for fcc hollow sites with a probability of 0.72, compared to 0.26 for on‐top sites.

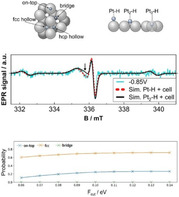

## 1 Introduction

**FIGURE 1:**
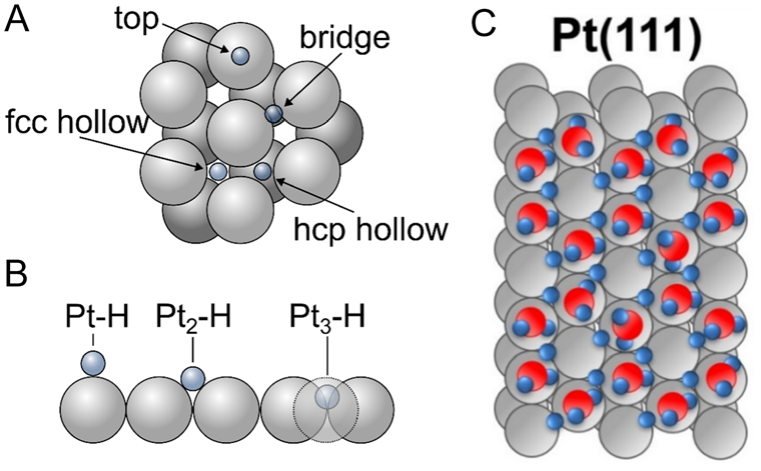
Schematic representation of typical adsorption sites at fcc‐metal surfaces. (A) Adsorbed hydrogen and (B) the number of coordinated Pt‐atoms at the surface of (C) Pt(111) close to the onset potentials of HER, according to theoretical DFT calculations and static model consideration. The H‐species occupy bridge (coordinated with two Pt atoms) and 3‐fold hollow sites (coordinated with three Pt atoms), with the top sites (coordinated with one Pt atom) remaining unoccupied according to such approaches. Blue is used for hydrogen, and red is used for oxygen atoms. Adapted from refs. [15, 16, 17].

Proton electroreduction, hydrogen oxidation reaction (HOR), and hydrogen evolution reaction (HER) are the model processes in modern electrocatalysis that enable a better understanding of the reaction mechanisms at electrified interfaces and develop advanced electrochemical surface science methodologies [1, 2, 3, 4, 5, 6]. A significant number of theoretical and experimental works have been performed in the past and recently to investigate the dominating reaction mechanisms and the nature of active centers for these reactions, e.g., [7, 8, 9, 10, 11]. However, one main complication of HER/HOR is the high mobility of reaction intermediates, namely, adsorbed atomic hydrogen. Therefore, these atomic species cannot be easily visualized using microscopies such as electrochemical scanning tunneling or atomic force microscopies under reaction conditions [12, 13, 14, 15]. The majority of the spectroscopies usually confirm just the presence of the H‐species but not their average location (cf., Figure 1A).

Several static density functional theory (DFT) calculations have shown that at 0 K, either in ultrahigh vacuum (UHV) or under “ice‐like” water layers, the 3‐fold coordinated fcc hollow site is the most energetically favorable adsorption site for atomic hydrogen [16, 18, 19, 20, 21, 22]. To more accurately account for the effects of thermal motion and solvent interactions on hydrogen adsorption at the Pt/H_2_O interface, multiple computational studies employing ab initio molecular dynamics (AIMD) simulations have been performed [23, 24, 25, 26, 27]. On the other hand, conclusions about the nature of HER active sites drawn from MD simulations are not always straightforward. For example, Le et al. found that snapshots from the AIMD trajectories revealed a lateral distribution of adsorbed hydrogen on the Pt(111) surface without any distinct pattern, except that the hydrogen adatoms predominantly populate the on‐top and 3‐fold hollow sites [25]. However, according to other AIMD simulations, both Mateo et al. and Ishikawa et al. report that, after the dissociation from hydrogen molecules, hydrogen adatoms are strongly bonded at the bridge site on the Pt(111) surface [28, 29]. Experimental infrared‐visible sum‐frequency generation data, however, suggest only on‐top adsorption [30]. Other laboratories report controversial conclusions on the adsorption centers, as summarized in [31].

The adsorption of hydrogen species at the surface of platinum occurs at more positive potentials than the HER potential, which is identical to the Volmer step in hydrogen evolution, often being considered the rate‐determining step [10, 32]. Therefore, elucidating the nature of the adsorption sites in that potential region (the so‐called hydrogen underpotential deposition region or in short: H_UPD_) is crucial to understand both the HOR and HER.

Therefore, this work introduces an in situ methodology that complements the electrochemical investigation of the H_UPD_ region with electron paramagnetic resonance (EPR) spectroscopy. In EPR, the application of an external magnetic field compels unpaired electrons to align either parallel (spin up) or antiparallel (spin down) with respect to the magnetic field, causing their energy levels to split.

**FIGURE 2:**
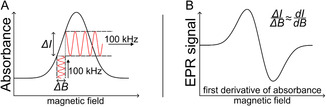
(A) Modulation of the magnetic field allows the detector to record a signal only at the modulation frequency, increasing the signal‐to‐noise ratio. (B) This produces a close approximation of the first derivative of absorbance.

Such electrons can change their spin state by absorbing or emitting a photon in the microwave range. As electrons are more likely to be found in a lower energy state, this results in an overall absorption of microwave radiation in the EPR chamber, which is detected as the EPR signal. The signal‐to‐noise ratio is generally improved by modulating the static magnetic field by 100 kHz, allowing the detector to measure only signals with the same modulation frequency, effectively recording the first derivative of the absorbance, as shown in Figure 2A,B. Additionally, unpaired electrons bound to atoms are coupled to the spins of the nearby atomic nuclei. This leads to hyperfine splitting of the electron energy states if the atomic nuclear spin is not zero. From the adsorption modes, it follows that there are different possibilities for the number of atoms coordinated with a hydrogen atom, as illustrated in Figure 1B.

As mentioned earlier, the role of atomic hydrogen, e.g., within HER, is crucial as the proton reduction or dissociation of H_2_O into an adsorbed hydrogen atom (H_ads_) and a hydroxide ion (Volmer step) precedes the formation of molecular hydrogen (H_2_) in both HER pathways. In the Volmer–Tafel mechanism, two adsorbed hydrogen atoms desorb as H_2_ (Tafel step), whereas the Volmer–Heyrovsky mechanism sees H_ads_ recombining with a solvent H_2_O molecule forming, e.g., H_2_ and OH^−^ (Heyrovsky step). Usually, the initial Volmer step is rate‐determining [33, 34], and therefore a link between adsorption sites and the current density is needed to optimize the catalyst surface for the Volmer step.

**FIGURE 3:**
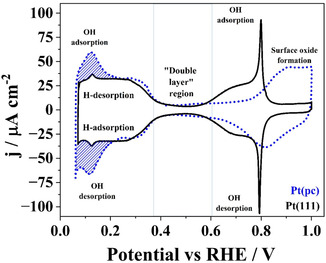
Comparison between cyclic voltammograms of bulk Pt(pc) (dotted blue line) and bulk Pt(111) electrodes (solid black line) in Ar‐saturated 0.1 M HClO_4_, measured with a scan rate of 50 mV/s.

Within the H_UPD_ adsorption region, the catalyst/electrolyte interface undergoes a potential‐dependent restructuring, ultimately leading to an increasing concentration of adsorbed hydrogen, which culminates in the formation of molecular hydrogen [35]. Single‐crystal surfaces are typically investigated to study these elementary reactions from which it becomes clear through cyclic voltammetry (CV) that the position of redox peaks is specific to basal planes [36, 37]. Because thick metals severely attenuate EPR signals and the geometry is limited to a 0.8 mm diameter, this study uses thin Pt(pc) wire in place of single‐crystal samples. Electrochemical measurements, in addition to X‐ray diffraction (XRD), reveal that polycrystalline platinum mainly consists of large (111)‐oriented surfaces besides higher index planes such as (331) [17, 38]. CVs that compare the here used Pt(pc) wires with Pt(111) single crystals show similarities, especially for potentials below 0.6 V versus RHE (Figure 3) and XRD data favors (111) and (311) orientation (cf., Figure S1). Additionally, the surface of the Pt(111) single crystal is characterized via STM (cf., Figure S2).

As EPR is sensitive to paramagnetic centers only, this in situ electrochemical EPR (EC‐EPR) study probes the possibility of paramagnetic intermediates within the H_UPD_ region. Protons (H^+^) are not paramagnetic, but once complexed with Pt after the Volmer step, the adsorbed, now neutral hydrogen atom becomes EPR active, and due to the minimal OH^−^ adsorption at H_UPD_ potentials, the only likely paramagnetic HER intermediate in the system [39]. While the EPR‐active nature of Pt_x_–H complexes has already been studied for some time as stable defects within silicon [40, 41] or in zeolite [42, 43], no literature is present for directly adsorbed hydrogen on transitional metal catalysts.

Usually, the lifetime of radicals or transient intermediates, along with general thermal motion, results in a diminishing signal at higher temperatures. Additionally, due to the temperature‐dependent spin–spin relaxation and, respectively, short relaxation times, the signal may undergo line broadening beyond detection, which is why experimental temperatures are typically within the cryogenic range. However, an investigation conducted at room temperature is crucial to gain an appropriate understanding of the HER under normal thermal conditions. Hence, this study investigates in situ measurements without the use of cryogenic temperatures and relies solely on potentiostatic holding.

## 2 Results

### 2.1 Simulation of EPR Signals and In Situ EPR Measurements

**FIGURE 4:**
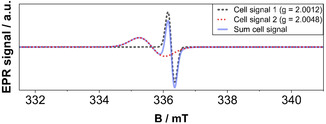
Simulation of cell signals close to *g* = 2.0023 and the resulting cell background.

To get an appropriate understanding of the paramagnetic nature of the whole in situ cell, several measurements of each part of the cell or at different conditions evaluate the EPR response of the cell. Without the electrolyte, the remaining EPR tube, wiring, and Pt wires produce two signals close to the free electron value of *g* = *g*_e_ = 2.0023: one broad component at *g* = 2.0048 and a narrow signal at *g* = 2.0012, which we attribute to the capillaries as well as to the Pt wire. Both signals are simulated and represent a cell background spectrum (Figure 4).

**FIGURE 5:**
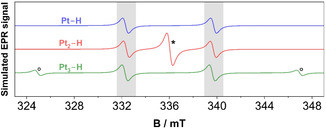
Simulation of hyperfine splittings of hydrogen in the vicinity of one, two, or three Pt atoms (Pt–H, Pt_2_–H, and Pt_3_–H). Gray areas mark the position of the satellite peaks, asterisk the additional center signal for Pt_2_–H, and circles (°) the outer pair of peaks for Pt_3_–H.

**FIGURE 6:**
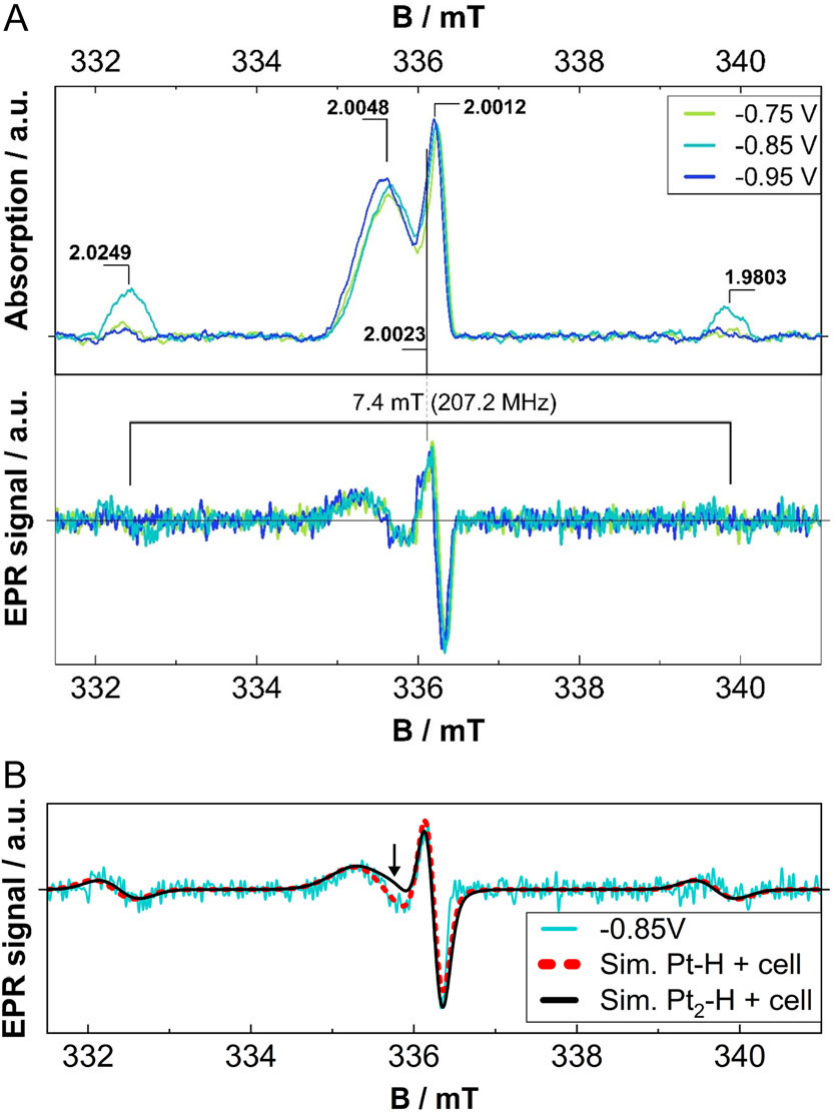
(A) Absorption spectra (top) from the recorded EPR spectra (bottom) with marked distance between the two hyperfine signals as well as their center. (B) The EPR spectrum at −0.85 V versus Pt and simulations for Pt–H and Pt_2_–H. The arrow marks the main deviation between the experimental and simulated data for Pt_2_–H.

To link possible hydrogen adsorption modes to the observed hyperfine splitting, EasySpin [44, 45] simulations of Pt–H, Pt_2_–H, and Pt_3_–H can be seen in Figure 5. The goal is not yet to identify the exact species but to rule out or assess the likelihood of individual adsorption modes. The simulated spectra are adjusted to match the satellite peak intensities (see gray areas in Figure 5), which have been observed in the measurement with a splitting of 207.2 MHz (Figure 6A). Whereas a single Pt atom leads to a splitting into two signals, symmetric to *g* = 2.0023, with the same intensity (1:1), Pt_2_–H produces an additional center signal with an intensity ratio of 1:2:1. And for Pt_3_–H, the four peaks share a 1:2:2:1 intensity distribution.

Since the signals of the satellite peaks are already subtle, the outermost Pt_3_–H peaks (see ‘**°**’ marks in Figure 5) are unlikely to be visible, and a broader scan also did not show them. In that sense, Pt–H and Pt_3_–H are indistinguishable, and only a smaller B‐range (331.5–341 mT) is investigated, which improves the signal‐to‐noise ratio for the given scan time of 120 s. Additionally, as only the platinum isotope ^195^Pt possesses a nuclear spin of nonzero (½), and its abundance is 33.8%, the chances for configurations with more ^195^Pt atoms became increasingly unlikely. The probability of having two ^195^Pt for Pt_2_–H is 11.4% and for three ^195^Pt in Pt_3_–H, the chance decreases to 3.9%. That means while EPR‐active 3‐fold sites may be too scarce, on‐top and bridge sites should still be detectable.

As shown in Figure 6A, the hyperfine satellite peaks are most pronounced at −0.85 V versus Pt and have nearly no intensity at other potentials. Interestingly, the lines from −0.85 V versus Pt appear to be slightly shifted toward higher fields. Both observations indicate a change on the electrode surface toward high negative potentials.

A recent computational study has indicated that at low coverages in the H_UPD_ region, hydrogen is adsorbed on fcc sites, but at more reductive potentials (overpotential deposition: H_OPD_ region), on‐top sites become predominant. This change is associated with the surface changing from hydrophobic to strongly hydrophilic, water reorientation, and a rapid transition to hydrogen coverages up to 0.9 ML. While the type of adsorption site is potential‐dependent, both types of adsorption sites coexist, and our measurement at −0.85 V versus Pt appears to be near this tipping point. Initially, the surface coverage increases, but at more negative potentials, EPR‐inactive molecular hydrogen is produced (cf., Figure S4) [46].

The comparison between the total simulated spectrum for Pt–H (also representative of Pt_3_–H) and Pt_2_–H for the most pronounced satellite peaks intensity at −0.85 V versus Pt shows good overall agreement between simulation and experimental data (Figure 6B). However, the central signal of Pt_2_–H at *g* = 2.0023 would lead to a deviation from the recorded spectrum, while the interaction between a single Pt atom and hydrogen—and, for that matter, any uneven number of Pt atoms—leads to symmetric line splittings (cf., Figure 5).

That means, even numbers of Pt atoms corresponding to 2‐fold bridge sites with their central line at *g* = 2.0023 seem unlikely to have contributed to the observed spectrum, whereas experimental data and simulations would agree with on‐top and 3‐fold sites.

This finding is in line with a recent DFT study, which has shown that on Pt(111), both fcc‐hollow and on‐top sites are free energy minima for hydrogen coverages ranging from *θ* = 0.167 to *θ* = 0.500, where the fcc hollow site is preferred over on‐top sites. In the following, this DFT framework is adapted to the experimental conditions of H_UPD_ close to HER, to yield MD simulations that further approach the theoretical maximum hydrogen coverages on Pt(111).

### 2.2 AIMD Simulation of High‐Coverage Pt(111) Surface

The in situ EPR measurements performed reveal that the most frequently occupied hydrogen (H) adsorption sites on the Pt(111) surface are the on‐top and 3‐fold hollow sites. Our previous AIMD simulations, combined with the corresponding laterally resolved free energy surface (LR‐FES), confirmed that only two basins for stable H‐adsorption sites exist on the Pt(111) surface in contact with an aqueous environment at room temperature and low coverage: the fcc hollow and on‐top site [23]. Experimental observations and computational analysis consistently indicate the presence of an upper limit of approximately 0.66 monolayer (ML) for H adsorption on the Pt surface [47, 48].

Our previous MD calculations were limited to hydrogen coverages up to 0.5 ML, leaving a gap in the LR‐FES of hydrogen between half an ML and the upper coverage limit. To this end, we compute the LR‐FES for H‐adsorption on the Pt(111) surface at a hydrogen coverage (i.e., 0.583 ML) above 0.5 ML but below the upper limit coverage in the presence of explicit water molecules.

**FIGURE 7:**
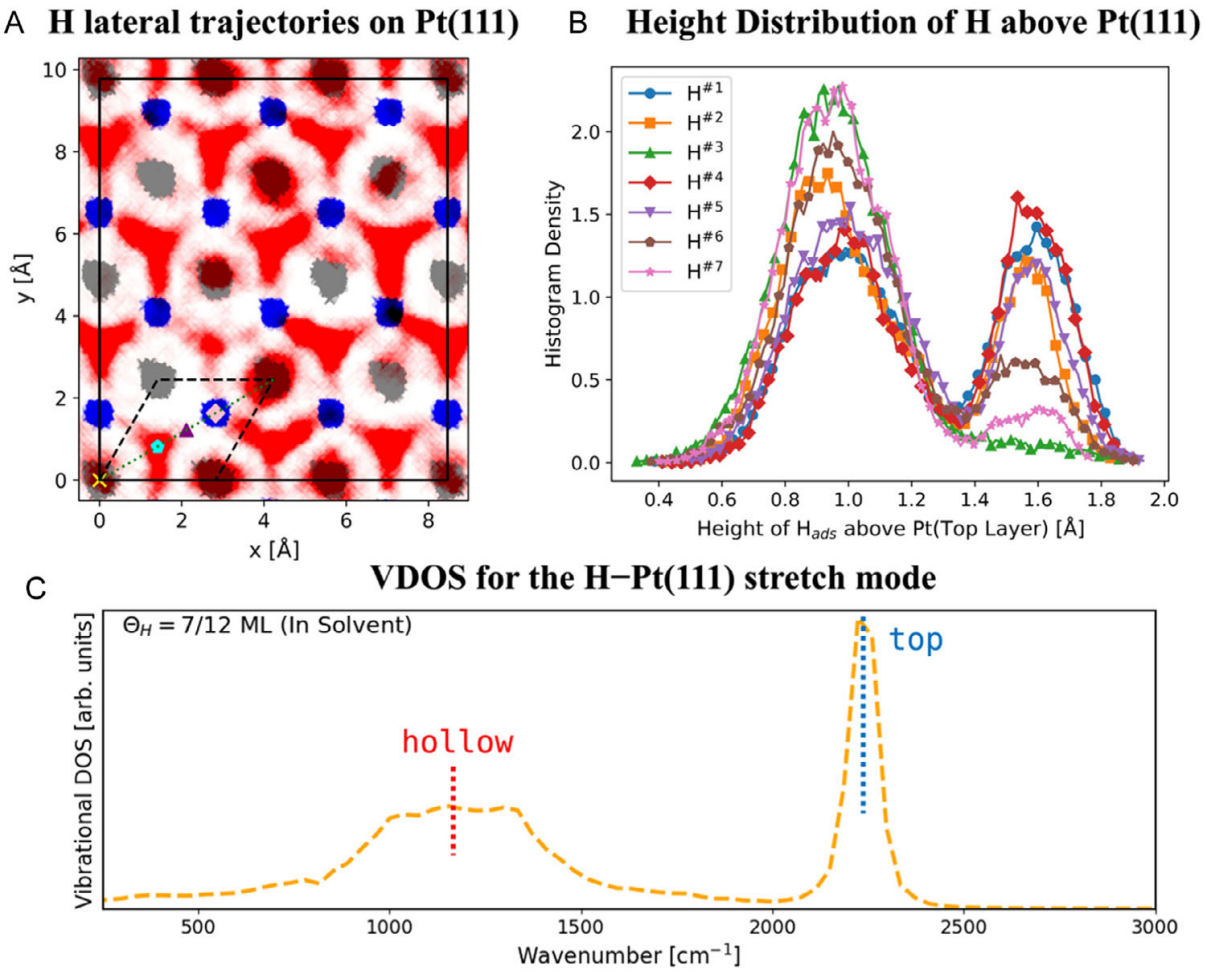
(A) Projected two‐dimensional (lateral) trajectories of adsorbed H atoms (red point cloud) and Pt atoms from the top (gray) and subsurface (blue) layers obtained from the AIMD simulations. The high‐symmetry adsorption sites are indicated by distinct markers: a yellow cross for the on‐top site, a cyan pentagon for the fcc site, a purple triangle for the bridge site, and a pink diamond for the hcp site. The dashed black parallelogram indicates the primitive (1 × 1) surface unit cell. (B) The height density distribution (a statistical counting of the height for distinct H‐atoms across different time intervals during the MD simulation) of all H‐adsorbates above the Pt surface. Note H^#i^ (*i* = 1–7) indicates different H atoms on the surface with an overall coverage of 0.583 ML. (C) The H–Pt vibrational power spectrum DOS for H at 0.583 ML in solvent.

Figure 7A presents the lateral trajectories of H atoms adsorbed at a coverage of 7/12 ML (0.583 ML) on the electrode surface, alongside the trajectories of Pt atoms from the first two surface layers, all mapped in two dimensions parallel to the surface plane. Compared to the trajectories of surface Pt atoms, the trajectories of H‐adsorbates span a wider surface area, underscoring the significant mobility (i.e., thermal motion) of H‐adsorbates on the Pt surface at room temperature in water. The adsorption sites with dense point clouds of H‐trajectories are primarily located at the on‐top and fcc hollow sites on the surface, with the trajectory patterns exhibiting sphere‐like and triangular configurations, respectively.

Consistent with our interpretation of the EPR measurements, the adsorption of H‐atoms at the 2‐fold bridge site is hardly ever observed in the AIMD simulation. Additionally, the trajectories suggest a higher probability for H‐adsorption at the 3‐fold fcc site compared to the on‐top site. This observation is also supported by the height distribution (i.e., the distance between the adsorbed H‐atoms and the top Pt layer) pattern of H‐adsorbates on the surface. The plot in Figure 7B shows two distinct peaks (positioned at approximately 0.9 and 1.6 Å) for the height of the H adsorbates above the surface. Based on the previous study, the first peak with a higher summit primarily corresponds to fcc adsorption on the surface, while the lower peak denotes on‐top adsorption, indicating a higher frequency of H‐adsorption at fcc sites [23]. The total vibrational density of states (VDOS) for the H‐Pt(111) stretch mode in Figure 7C further validates our AIMD simulation results with the reported surface‐enhanced infrared reflection absorption spectroscopy (SEIRAS) measurement and previous theoretical investigations: the broad feature centered around 1100 cm^−1^ is attributed to H adsorption at the surface hollow site, while the prominent peak observed near 2200 cm^−1^ corresponds to H adsorbed at the on‐top site [23, 25, 49].

**FIGURE 8:**
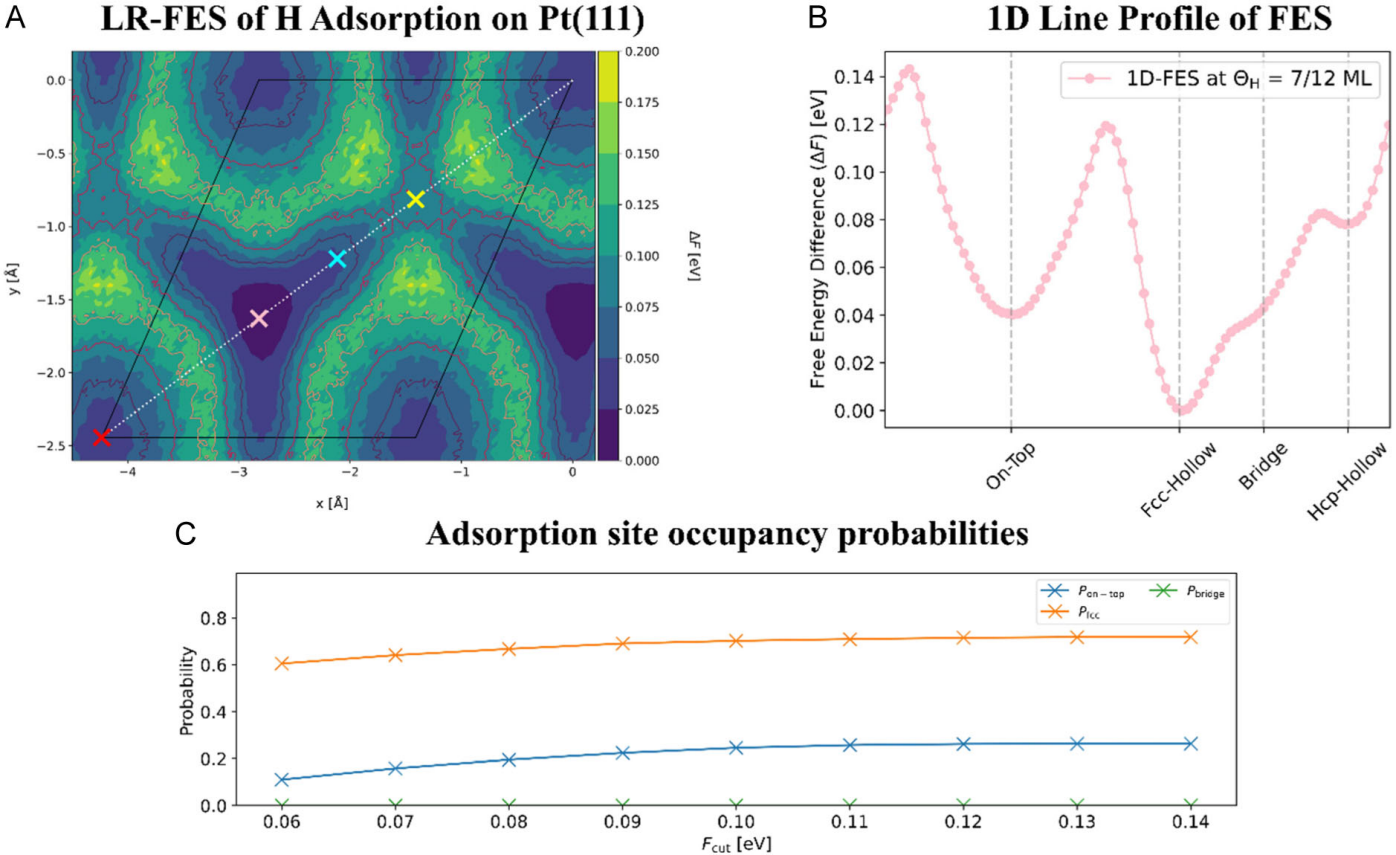
(A) LR‐FES of H‐adsorption on the Pt(111) surface within the primitive (1 × 1) surface unit cell. The colored crosses (red, pink, cyan, and yellow) in the figure represent the on‐top, fcc, bridge, and hcp adsorption sites, respectively. (B) 1D line profile of the H‐adsorption free energy surface at 0.583 ML coverage along the diagonal pathway (indicated by the dotted diagonal line in (A)), highlighting the high‐symmetry adsorption sites. (C) Convergence of site occupancy probabilities (P) as a function of the free energy cutoff (F_cut_).

The two‐dimensional Helmholtz free energy profile of the H‐adsorbates on the Pt surface was computed to better understand the origin of the H lateral trajectories (i.e., adsorption pattern) on the Pt surface. Figure 8A shows the laterally resolved free energy surface of H‐adsorption at a coverage of 0.583 ML on the Pt(111) surface in contact with water. The free energy surface reveals two distinct symmetry‐inequivalent free energy basins for H‐adsorption, indicating the (meta)stable adsorption sites on the electrode surface as the fcc hollow site and the on‐top site. The H adsorption free energy at the 3‐fold fcc hollow site is obviously the global minimum among all the adsorption sites on the Pt(111) surface. This aligns with the observation of the densest point cloud concentrated at the fcc sites in the lateral trajectories plot. The free energy surface of H adsorption at a coverage of 0.583 ML in this work exhibits a strong similarity to our previous results for lower coverages, ranging from 0.167 to 0.50 ML, indicating that the hydrogen coverage has a minimal impact on the overall free energy surface [23].

In Figure 8B, the line profile of the free energy surface (FES) along the diagonal (dotted diagonal line in Figure 8A) of the primitive surface unit cell illustrates the H‐adsorption pathway traversing the high‐symmetry adsorption sites on the surface. Three distinct local minima for H adsorption energy are identified: the 3‐fold fcc hollow, the on‐top, and the 3‐fold hcp hollow site. In contrast, no local minimum is observed near the bridge adsorption site, excluding this 2‐fold site as a (meta)stable adsorption location for H‐atoms on the Pt(111) surface.

The computed FES for H Adsorption in this study also shows good correlation with previously reported static DFT calculation results. Consistent with our findings, Källen et al. also identified the 3‐fold fcc hollow site as the minimum energy site for hydrogen adsorption at Pt(111) surface based on direct DFT total energy calculations. The qualitative correspondence between the FES and the static DFT‐calculated energy surface may be expected, since the Gibbs free energy of H adsorbed on the metal surface can be reasonably approximated as being linearly correlated with the DFT‐calculated adsorption energy. Nevertheless, residual quantitative discrepancies persist between the two methodologies. While static DFT‐computed minimum energy surface ranks the sites as fcc < hcp < bridge < ontop, our FES obtained from AIMD calculations reveals a different order: fcc < ontop < hcp (note that the bridge site is excluded as it does not correspond to a local minimum). This FES ordering aligns more closely with experimental observations, indicating the importance of incorporating entropy and solvent effects into DFT calculations to accurately describe hydrogen adsorption site preferences.

Figure 8C depicts the convergence of adsorption site occupancy probabilities for the on‐top, bridge, and fcc sites as a function of the free energy cutoff (*F*_cut_). This cutoff is used to classify a spatial point as part of a specific adsorption site, based on the condition *F*(*r*) ≤ *F*_cut_. The converged occupancy probabilities for the fcc, on‐top, and bridge sites are approximately 0.72, 0.26, and 0, respectively, for a coverage of 0.583 ML on the Pt(111) surface. This reaffirms the interpretation of the experimental EPR spectra (as shown in Figure 6), which attributes the signals predominantly to on‐top (Pt–H) and possibly 3‐fold fcc/hcp (Pt_3_–H) adsorption sites.

## 3 Conclusions

For the first time, EPR spectroscopy was used to probe the electrosorption of hydrogen in the H_UPD_ region at polycrystalline platinum electrodes in acidic electrolytes. From our EPR measurements close to room temperature, it is hypothesized that the most frequent positions of the adsorbed H‐species are on‐top and hollow sites, consistent with our accompanying ab‐initio AIMD simulations. Bridge sites appear to be much less likely to be occupied by H^0^, contrary to other AIMD observations.

After consideration of alternative causes of the observed signal, e.g., OH^−^ or transient organic radicals—both of which are unlikely to contribute, as within the H_UPD_ region OH^−^ concentration is negligible, and the extended potential holds render temporary radicals unlikely; our EPR measurements suggest adsorbed atomic hydrogen in situ at the surface of platinum electrocatalysts. In combination with the respective DFT calculation, this study should be particularly interesting for HER electrocatalysis methodology development, and for pointing toward the level of realism one must strive for in the used theoretical quantum chemistry (e.g., DFT) models.

## 4 Experimental and Methodology

### 4.1 EPR Spectrometer

The SpinscanX CW‐EPR spectrometer (LINEV ADANI, Belarus) with 10^14^ spins/T sensitivity was used for the combined electrochemical and EPR experiments. A modulation frequency of 100 kHz was kept unchanged for all experiments for the 9.418 GHz microwave frequency. All measurements were performed at room temperature. The modulation amplitude is 200 µT, and the microwave power was set to 0.3 mW for all measurements.

### 4.2 In Situ Cell Assembly

**FIGURE 9:**
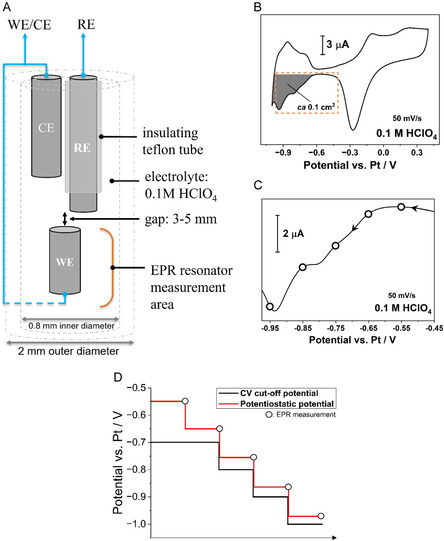
(A) Schematics of the EC‐EPR cell (WE, working electrode; RE, reference electrode; CE, counter electrode). (B) Typical recorded cyclic voltammogram of the polycrystalline platinum electrode (dark gray surface area: ca. 0.1 cm^2^) in the EC‐EPR cell. (C) The cathodic scan of the same cyclic voltammogram (orange rectangle in (B)) in the H_UPD_ region with points indicating the potential holds at which in situ EPR spectra were recorded. (D) Change of negative potential limits during the in situ study.

Figure 9A shows the schematics of the capillary used for the in situ study. Due to the small inner diameter of the capillary (0.8 mm), conventional hydrogen reference electrodes (REs) are not feasible. Hence, the electrochemical cell uses 0.2 mm diameter Pt wires (MaTecK, 99.9%) for all electrodes, with the RE being covered with 0.36 mm inner diameter ETFE tubing (VICI) to electrically isolate it from the counter electrode (CE). All electrodes were soldered into their respective contacts, and silicone tubing with an inner diameter of 2.0 mm was added to the base of the electrodes with silicone glue (Elastosil E43, Wacker). This seals the quartz capillary (inner and outer diameters 0.8 and 2.0 mm, respectively) at both the bottom and top openings. As the surface area within the capillary is limited, the working electrode's effective surface area was increased by eroding the Pt wire submerged in 1 M NaNO_3_ (Merck) solution by applying large amplitude sinusoidal voltammetry (LASV) at ±10 V at 200 Hz for two 60‐s periods with one 30‐second observation period in air in between. The eroded Pt wire was subsequently rinsed with DI water and stored in electrolyte. A comparison between pristine and eroded wire is shown in Figure S5. The cells were subsequently assembled in Ar atmosphere glove box by preparing a 0.1 M HClO_4_ electrolyte (Merck), deaerated with Ar gas for 20 min, and injecting it with a syringe into the capillary. The assembled cell was airtight and was then moved from the glove box to the EPR chamber, where it was placed inside an outer tube, the bottom of which also housed a socket for the working electrode (WE) contact. Figure S6A illustrates the individual components of the cell, and the assembled cell in its housing is presented in Figure S6B.

### 4.3 CV

A VSP‐300 potentiostat from BioLogic (France) was used to perform cyclic voltammograms according to the following protocol: After assembly, the surface of the polycrystalline platinum wire was electrochemically cleaned by performing cyclic voltammograms (CVs) between −0.4 V and +0.4 V versus Pt until the CVs became stable. Then, the potential window was increased to −0.7 V to +0.4 V versus Pt and cycled until stable. For the in situ study, the potential range was successively extended to negative potentials using the following measurement protocol: For measurements at −0.55 V and −0.65 V versus Pt, the CV cutoff potential was maintained at −0.7 V versus Pt. For the remaining potential steps, the cutoff potential followed the reduction in potentiostatic potential (Figure 9B–D). As −0.55 V and −0.65 V versus Pt still lie within the double‐layer region, only measurements with potentials more negative than −0.65 V versus Pt are shown.

Before recording the EPR spectra, CVs with the respective limit were performed until the CVs were stable, followed by a potential hold at the same potential where the EPR measurement will take place, until the current remains constant (cf., Figure S7). This also ensures that no transient species, such as organic radicals, can temporarily produce a signal. As the electrolyte volume is limited within the capillary, and due to the lack of a proper RE, the applied potential may shift slightly during the potential hold.

### 4.4 EPR Measurements

At each potentiostatic step, several EPR spectra were recorded with 120 s sweep time each. Within the cavity, temperatures ranged from 43°C to 46°C during the entire recording period. The spectra at each potential were averaged, baseline‐corrected, and FFT‐filtered with a 25 Hz low‐pass filter. For the absorption spectra, the EPR signals are integrated and baseline‐corrected.

### 4.5 Simulated EPR Signals

The simulations of the signals were performed with the MATLAB library EasySpin. Also, based on the recorded data, the signals are assumed to be isotropic. The signals associated with the in situ cell itself, without the electrolyte, are represented by a narrow and a broad component (Figure 4). For the hyperfine splitting, either one, two, or three ^195^Pt nuclei (spin ½, abundance: 33.83%) were assumed to interact with a hydrogen electron with spin ½. Apart from using a Lorentzian lineshape for all signals, the weight was also adjusted so that each simulation fits the two satellite signals, which were registered additionally to the cell signature. The distance between the peaks is used to determine the hyperfine coupling constant of Pt–H, which is found to be 207.2 MHz. Both interaction matrices of Pt_2_–H (55 45 207.2; 55 45 207.2) and Pt_3_–H (370 25 220; 370 25 220; 370 25 220) are then adjusted to match the satellite peaks.

### 4.6 AIMD Calculation

**FIGURE 10:**
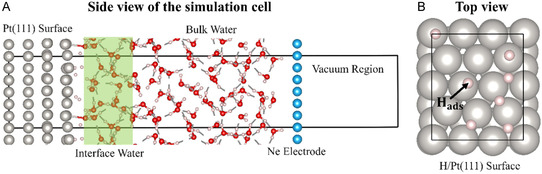
Visualization of the periodic supercells used to simulate the Pt(111) surface with 0.583 ML H adsorbed with the presence of explicit water: (A) side view (along the surface normal direction) of the simulation cell; (B) top view of the H adsorbed Pt(111) surface (the water has been removed for clarity). The spheres colored gray, red, white, and blue to represent Pt, O, H, and Ne atoms, respectively.

The simulation cell used for modeling the Pt(111) electrode in contact with water was a four‐layer ($3\times 2\sqrt{3}$) slab prototype, with each layer containing 12 atoms (Figure 10). H‐atoms (Θ_H_ = 7/12 ML ≈ 0.583 ML) were adsorbed onto fcc hollow sites on the top layer of the Pt electrode prior to initiating the AIMD simulation. A total of 64 explicit water molecules, pre‐equilibrated using classical MD with the TIP3P potential as implemented in the LAMMPS package, were positioned above the Pt surface [50, 51]. These water molecules were constrained by an overlying Ne electrode, following a setup similar to that described in our previous work [23]. The distance between the Ne CE and the Pt electrode was optimized to ensure accurate liquid water density at 300 K and minimize the free energy of the entire Pt/H_2_O/Ne sandwich system. A vacuum region of 12 Å was introduced along the *z*‐axis between the Ne electrode and the pristine side of the Pt surface. The lattice parameters of the whole unit cell were (8.46 × 9.77 × 46.05) Å^3^. The adsorbed H atoms, along with the first and second Pt layers adjacent to the water molecules, were allowed to relax during the simulation, whereas the Ne atoms and the remaining two Pt layers at the bottom of the slab were kept frozen.

All DFT calculations in this work were performed using the Vienna ab initio simulation package (VASP) [52, 53, 54]. The core electrons of Pt, O, and H were simulated with the projector‐augmented wave (PAW) method with a plane‐wave energy cutoff of 500 eV [55]. The revised PBE functional was employed for its demonstrated accuracy in reproducing water‐cluster formation energies, bulk liquid water structure, and interfacial properties, as well as for its improved electronic stability in Pt/water AIMD simulations [56, 57, 58, 59]. Van der Waals interactions were accounted for by treating the dispersion energy with the Grimme D3 type correction [60]. A (2 × 2 × 1) Monkhorst–Pack *k*‐point grid was used for all calculations involving the Pt/water interface to ensure the convergence of the H adsorption energy to 5 × 10^−2^ eV per unit cell [61]. For the AIMD simulations, water molecules and the Pt surface (with adsorbed H) were first equilibrated for 10 ps, after which an additional 30–40 ps of sampling was collected in the canonical ensemble with a Langevin thermostat at 300 K [62, 63]. The time step of the MD simulation was set to 0.9 fs. The number of H‐atoms being adsorbed on the Pt surface was monitored to ensure that no H‐desorption occurred (i.e., the adsorption coverage remained constant) throughout the AIMD simulation. The total vibrational density of states (VDOS) for the adsorbates was computed using the Welch periodogram method on the finite‐difference velocity of the H adsorbates along the surface normal direction [23]. All calculations and associated workflows presented here were automated by the pyiron package [64].

### 4.7 Hydrogen Adsorption Free Energy Profiles

Building on the method outlined in our previous work [23], the LR‐FES of H‐adsorption on the Pt surface was derived by the Boltzmann inverse from the two‐dimensional density of H‐adsorption, *ρ*(*r*_∥_), which indicates the probability of locating an adsorbed H‐atom at the lateral position *r*_∥_ [23]

$$F\left(r_{∥}\right)=-k_{B}T\ln[\frac{\rho \left(r_{∥}\right)}{\rho^{\max}\left(r_{∥}\right)}]$$

where *k*_B_ is the Boltzmann constant, and *T* is the temperature. *ρ* ^max^(*r*_∥_) refers to the maximum lateral density, ensuring that the free energy minimum of an adsorbed H‐atom at the electrode surface is set to 0 eV. This allows for the determination of the relative free energy differences of hydrogen adsorption across various adsorption sites on the Pt surface. The lateral density can be estimated from the lateral trajectories of adsorbed H‐atoms $\left(R_{∥}^{I_{H}}\left(t\right)\right)$

$$\rho (r_{\text{∥}})=\frac{\sum_{i_{H=1}}^{N_{H}}\sum_{\hat{\sigma }}\sum_{t=1}^{N_{t}}\delta (r_{\text{∥}}-\hat{\sigma }R_{\text{∥}}^{I_{H}}(t))}{N_{H}N_{t}}$$

with the Dirac delta function, *δ*, being employed to create a two‐dimensional histogram mapping the lateral positions of H‐adsorbates across a grid that covers the primitive unit cell of the Pt(111) surface. $\hat{\sigma }$ considers the point group and translational symmetry operators for the lateral positions within the *C*_3_ *_v_* space group of the investigated fcc (111) surface. *N_H_* denotes the total number of adsorbed H‐atoms (which in this case is *N_H_* = 7), while *N_t_* represents the time steps used in the AIMD simulations.

## Supporting Information

Additional supporting information can be found online in the Supporting Information section. **Supporting Fig. S1**: XRD patterns of the Pt(pc) wire. **Supporting Fig. S2**: STM pictures of Pt(111) single crystal in Ar atmosphere. The images A)‐C) were taken at different magnifications. The linescan reveals the surface roughness of 0.2 nm. **Supporting Fig. S3:** Absorption spectrum of the in‐situ cell without (gray) and with applied potential (green, teal and light blue color). **Supporting Fig. S4:** EPR spectra at selected H_UPD_ potentials. Gray dashed line marks the positions of the observed EPR signals. **Supporting Fig. S5:** SEM pictures of a pristine Pt(pc) wire before (A)‐(B) and after erosion (C)‐(D). **Supporting Fig. S6:** (A) Individual components of the in‐situ cell. (B) Complete cell inside the outer EPR glass tube with a wire connecting to the working electrode following its outer surface. **Supporting Fig. S7:** Illustration of the importance of potential holds. Absorption spectra after potentiostatic waiting at measured potentials (blue, red) and during stabilizing cyclic voltammograms (gray).

## Conflicts of Interest

The authors declare no conflicts of interest.

## Supporting information

Supplementary Material

## Data Availability

The data that support the findings of this study are available from the corresponding author upon reasonable request.
