# Supplementary material for: In Situ EC‐EPR Spectroscopy and DFT Analysis of HUPD on Polycrystalline Pt
Source: ChemSusChem. 2026 Mar 8;19(5):e202501908. doi: 10.1002/cssc.202501908 (PMC12967722; doi:10.1002/cssc.202501908)
Supplement: Supplementary file 1 — Supplementary Material [file CSSC-19-e202501908-s001.pdf]

In-situ EC-EPR Spectroscopy & DFT simulations of H_{upd} on Polycrystalline Pt

Rainer Götz,[(1),a] Kimmo Pyyhtiä,[(1),b] Bingxin Li,[(1),c] Theophilus K. Sarpey,[a,d] Kun-Ting Song,[a] Mira Todorova,[c] Nadezhda Kukharchyk,[e] Siegfried Schreier,[a] Pekka Peljo,[b] Elena L. Gubanova,[a] Jörg Neugebauer*[c], Aliaksandr S. Bandarenka*[a,f]*

[a] - *Physics of Energy Conversion and Storage, TUM School of Natural Sciences, Department of Physics, Technical University of Munich, James-Franck-Str. 1, 85748 Garching, Germany*

[b] - *Department of Mechanical and Materials Engineering, University of Turku, Vesilinnantie 5, 20500 Turku, Finland*

[c] - *Max-Planck-Institut für Eisenforschung GmbH Max-Planck-Str. 1, 40237 Düsseldorf, Germany*

[d] - *GSI Helmholtzzentrum für Schwerionenforschung GmbH, Planckstr. 1, 64291 Darmstadt, Germany*

[e] - *Walther-Meißner-Institute for Low Temperature Research, Bavarian Academy of Sciences and Humanities, Walther-Meißner-Str. 8, 85748 Garching, Germany*

[f] - *Catalysis Research Center TUM, Technical University of Munich, Ernst-Otto-Fischer-Str. 1, 85748 Garching, Germany*

(1) Authors contributed equally

Corresponding authors:

* E-mail: elena.gubanova@tum.de (E. L. Gubanova),

* E-mail: neugebauer@mpie.de (J. Neugebauer)

* E-mail: bandarenka@ph.tum.de (A.S. Bandarenka)

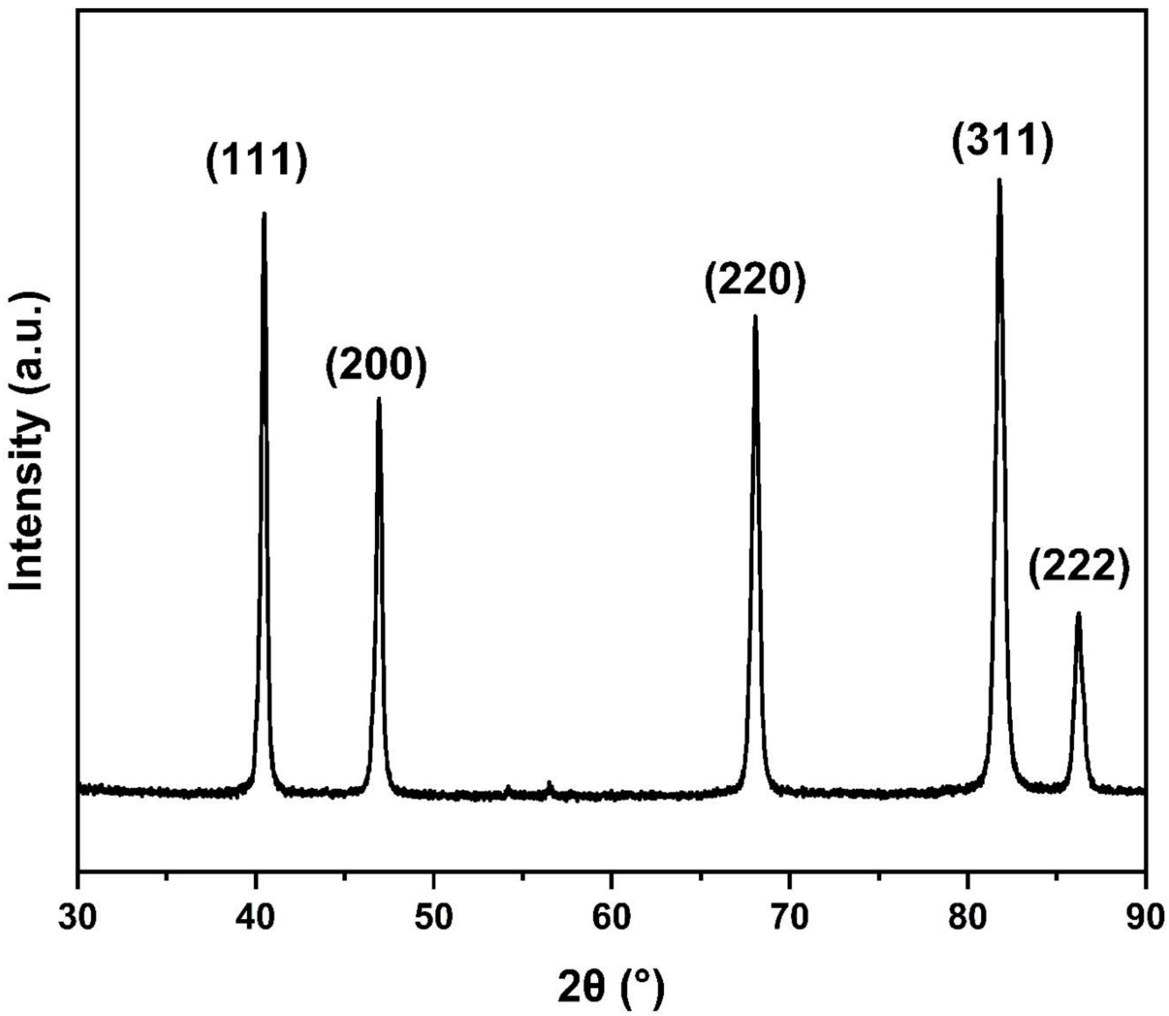

Figure S1. *XRD patterns of the Pt(pc) wire.*

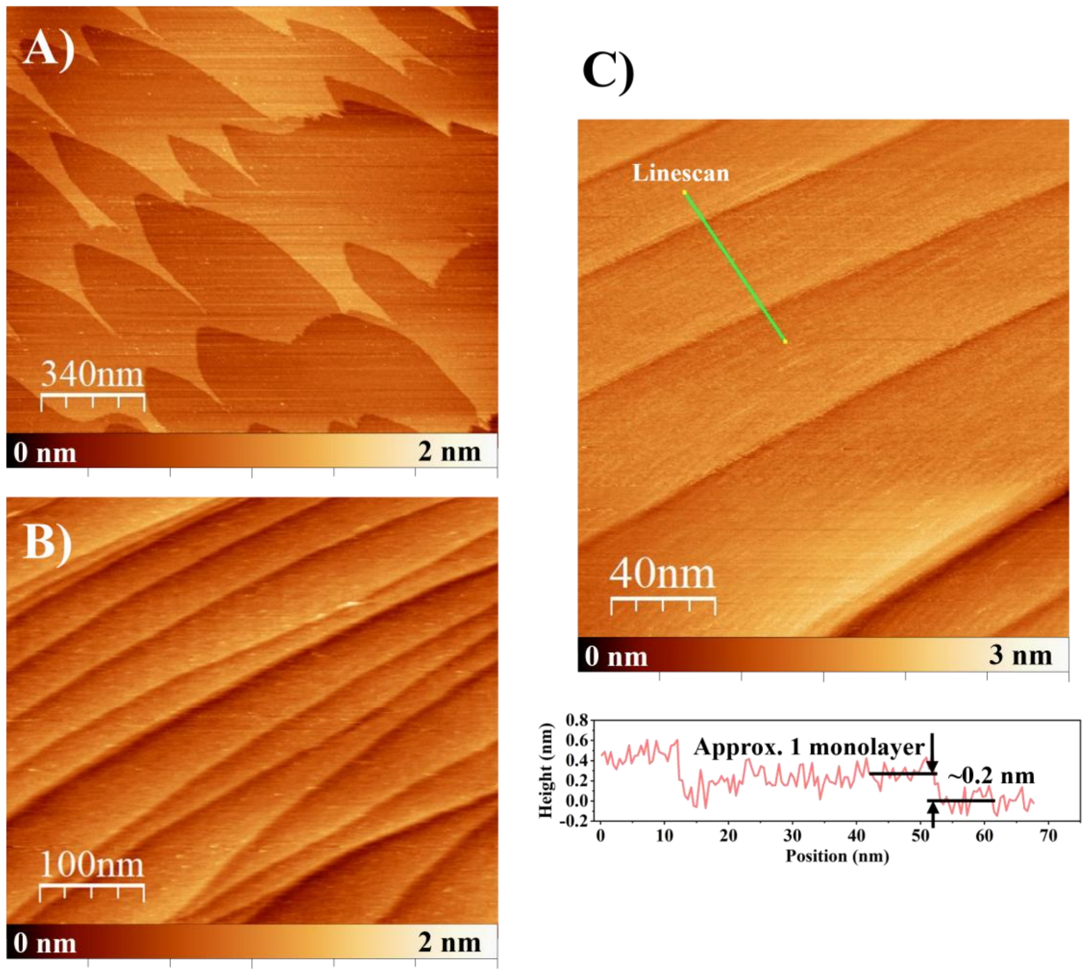

zFigure S2. *STM pictures of Pt(111) single crystal in Ar atmosphere. The images A)-C) were taken at different magnifications. The linescan reveals the surface roughness of 0.2 nm.*

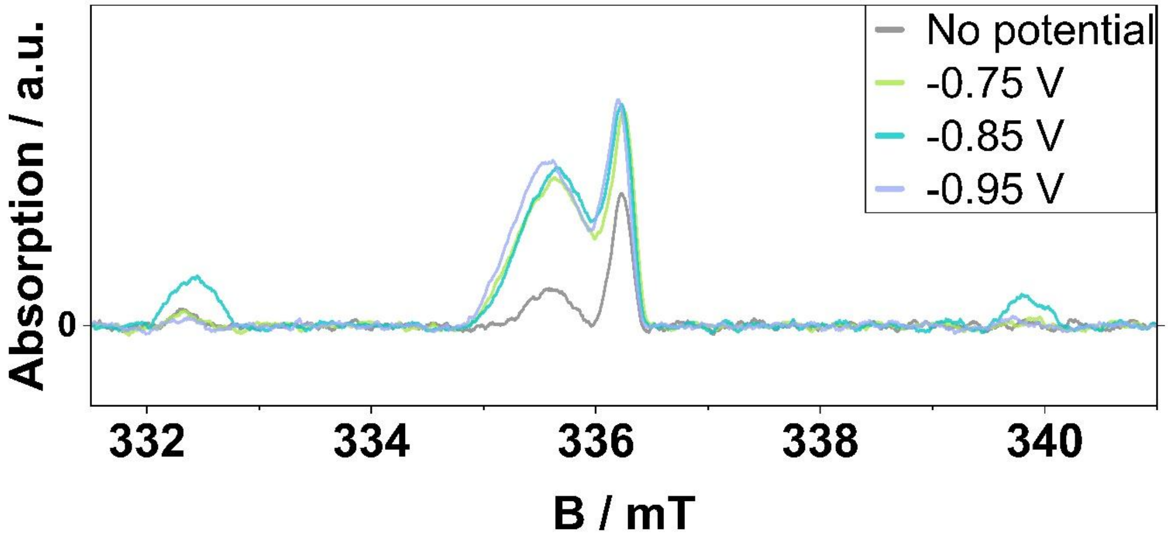

Figure S3. *Absorption spectrum of the in-situ cell without (gray) and with applied potential (green, teal and light blue color).*

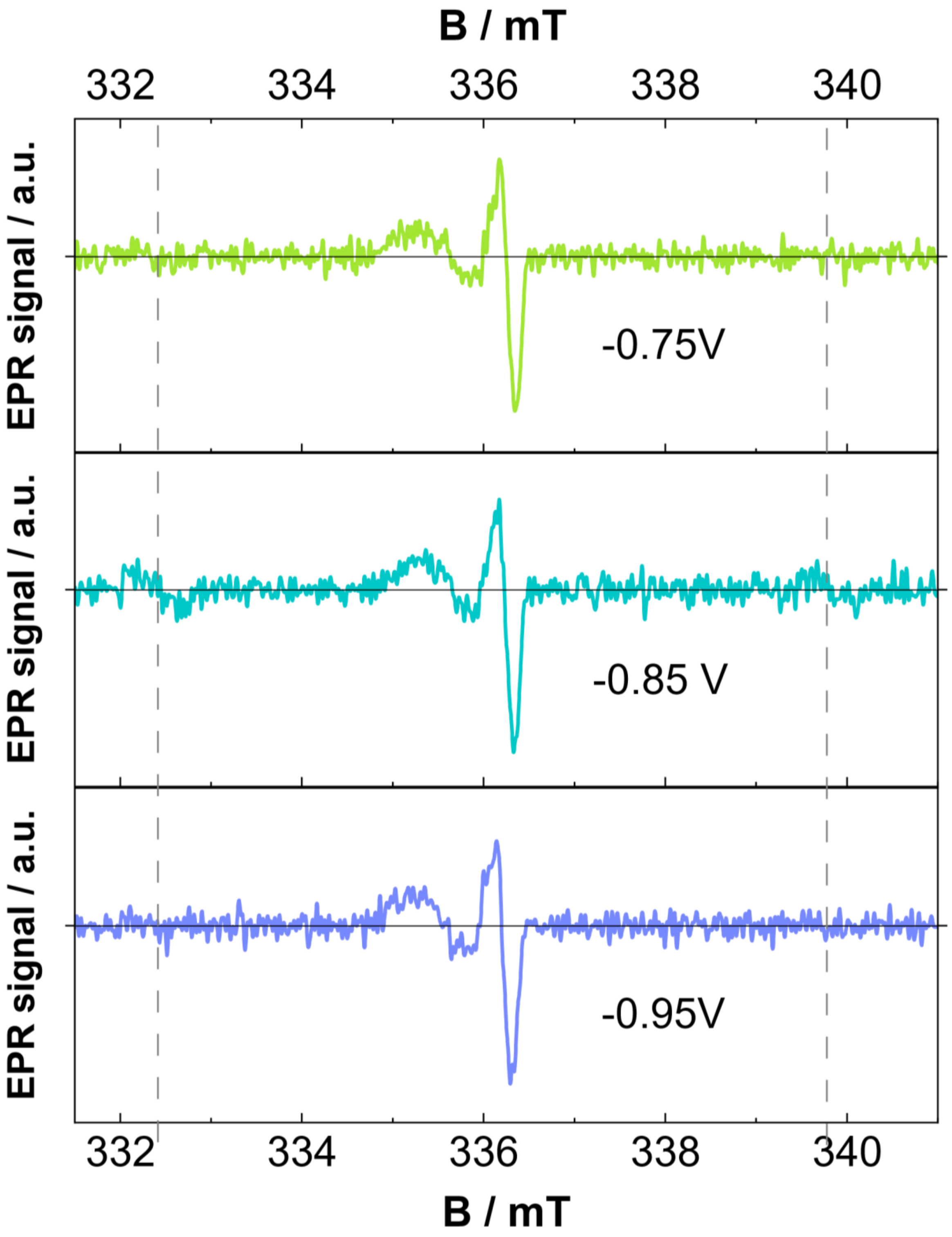

Figure S4. *EPR spectra at selected H_{UPD} potentials. Gray dashed line marks the positions of the observed EPR signals.*

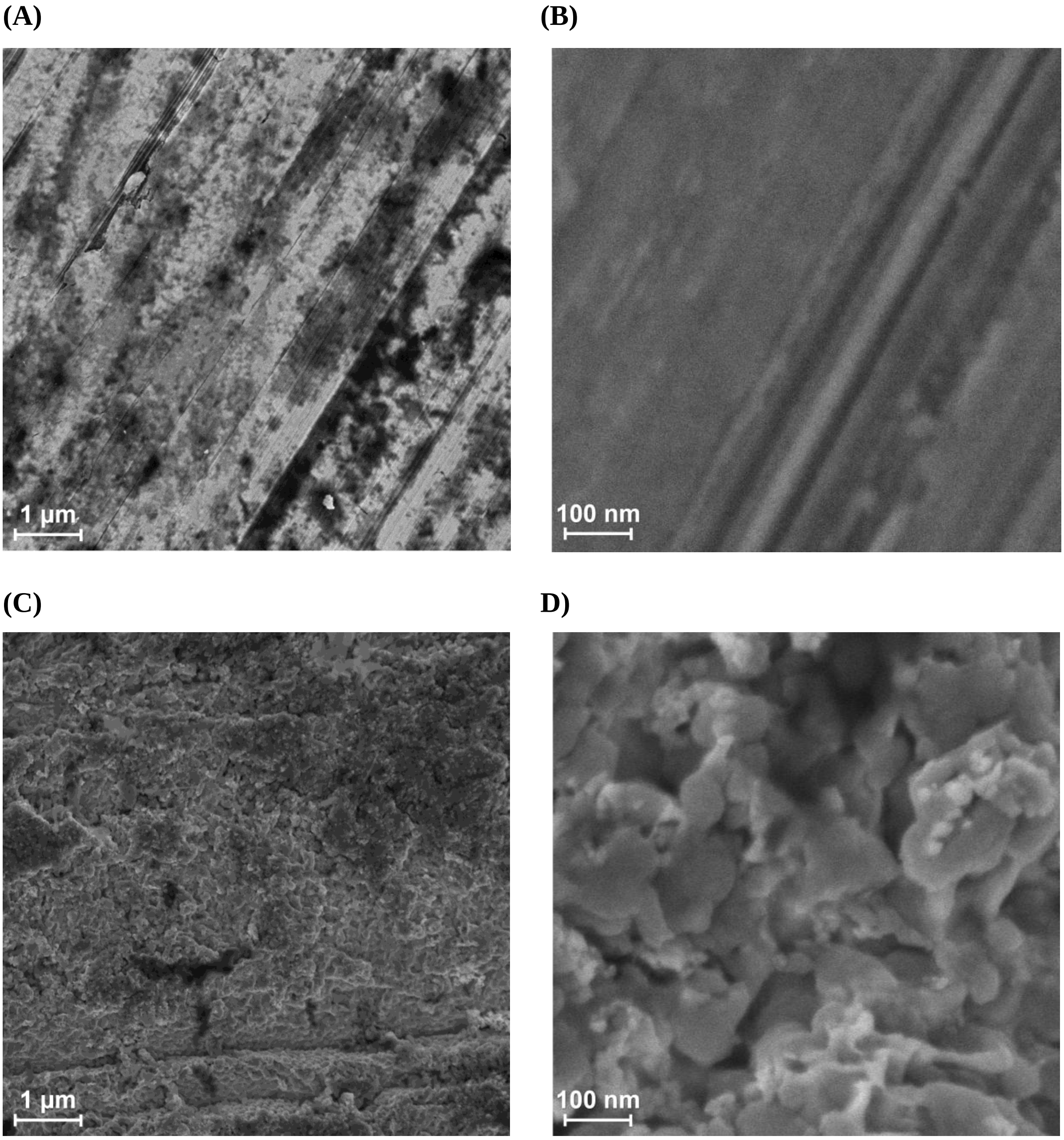

Figure S5. *SEM pictures of a pristine Pt(pc) wire before (A)-(B) and after erosion (C)-(D).*

A)

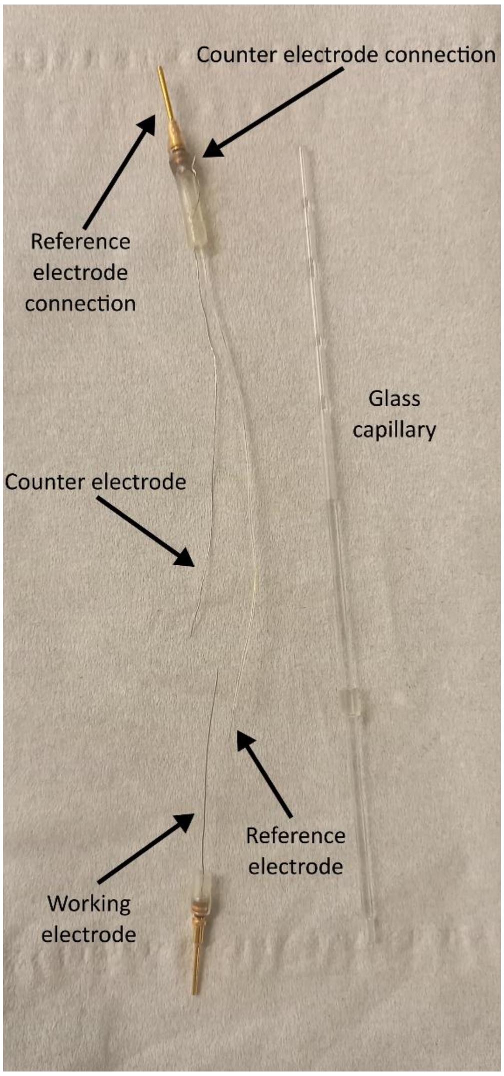

B)

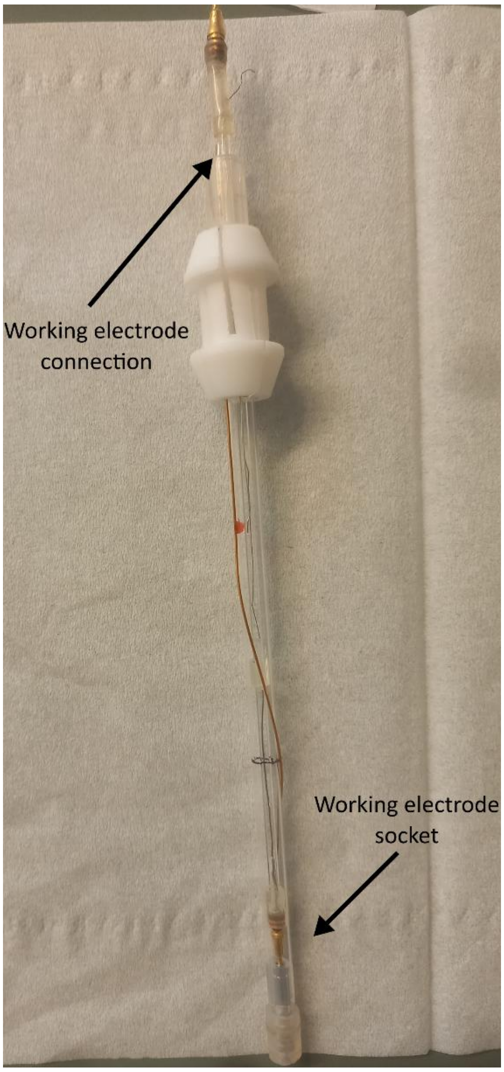

Figure S6. *(A) Individual components of the in-situ cell. (B) Complete cell inside the outer EPR glass tube with a wire connecting to the working electrode following its outer surface.*

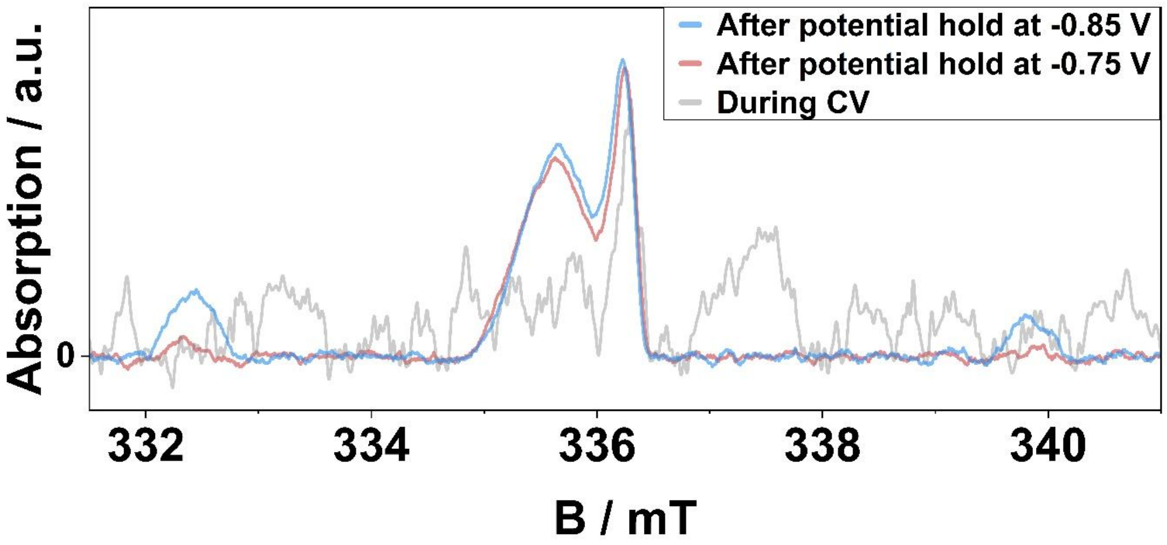

Figure S7. *Illustration of the importance of potential holds. Absorption spectra after potentiostatic waiting at measured potentials (blue, red) and during stabilizing cyclic voltammograms (gray).*
